# Multimodal optical clearing to minimize light attenuation in biological tissues

**DOI:** 10.1038/s41598-023-48876-x

**Published:** 2023-12-06

**Authors:** Behnam Shariati B. K., Mohammad Ali Ansari, Seyyede Sarvenaz Khatami, Valery V. Tuchin

**Affiliations:** 1 Laser and Plasma Research Institute, Shahid Beheshti University, Tehran, 19839 69411 Iran; 2 Science Medical Center, Saratov State University, 83 Astrakhanskaya Str., Saratov, Russia 410012

**Keywords:** Optics and photonics, Optical techniques

## Abstract

The biggest obstacle to optical imaging is light attenuation in biological tissues. Conventional clearing techniques, such as agent-based clearing, improve light penetration depth by reducing scattering, but they are hampered by drawbacks including toxicity, low efficiency, slowness, and superficial performance, which prevent them from resolving the attenuation problem on their own. Therefore, quick, safe, and effective procedures have been developed. One of them involves using standing ultrasonic waves to build light waveguides that function effectively in the tissue depth while minimizing scattering. Temporal optical clearing is another agent-free strategy that we introduced in our previous article. Whereas not deep, this technique minimizes both light absorption and scattering by pulse width variation in ultra-short pulse regime. Consequently, it can be a complementary method for ultrasonic optical clearing. In this work, we enhanced the light penetration depth in chicken breast tissue by 10 times (0.67–6.7 cm), setting a record in literature by integrating three clearing methods: agent-based, ultrasound-based, and temporal. Here, optical coherence tomography, Bear–Lambert, and fluorescence tests have been used to study the light penetration depth and optical clearing efficiency. Presented work is an essential step in development of diagnostic techniques for human body, from cells to organs.

## Introduction

The absorption and scattering of light in biological tissues has hampered optical imaging's depth and resolution. For example, high-resolution techniques such as optical coherence tomography (OCT) limit imaging to the tissue's surface layer. OCT is reliable down to a few millimeters with a resolution of a few microns, while photoacoustic tomography (PAT), with a resolution of a few hundred microns, observes a depth of a few centimeters of the tissue^1–3^, or diffuse optical tomography (DOT), which has a greater imaging depth (several centimeters), has a much lower resolution (a few millimeters)^4^. Conventional optical clearing techniques aim to decrease scattering and improve image's depth and resolution. Agent-based optical clearing techniques minimize light scattering in various ways. Examples of how optical clearing agents (OCAs) works include dissociating collagen fibers, matching the refractive index of tissue components and interstitial fluid, and tissue dehydration. There are several OCA-based clearing strategies described in references^5–11^. These publications examine how optical clearing may improve optical imaging's resolution and penetration depth while also boosting the stability of the tissue's optical characteristics after clearing, presenting novel clearing solutions, and exploring optical clearing in new types of tissues. Tissue shrinkage, local hemostasis, and the toxicity of certain OCA are some of the drawbacks of agent-based procedures. Furthermore, occasionally utilizing OCAs resulted in skin irritation or inflammation, which naturally recovered fast^11,12^. Glycerol, one of the most common OCAs, for example, can change the shape of the skin by separating the collagen fibers. It has been demonstrated that anhydrous glycerol significantly affects the cutaneous vasculature^13,14^. This issue raises concerns about the use of clearing chemicals in in vivo imaging, leading researchers to use safer clearing approaches. Because OCAs require a considerable amount of time to diffuse throughout the tissue, agent-based techniques are not a fast way to clear tissue. The impact of an OCA on imaging has been examined in various research. Several studies show that the effect of an OCA on image quality might take anywhere from a few minutes to several hours^5–18^. An important factor is that some OCAs only diminish part of the scattering. This topic has been covered both theoretically and practically in the Refs.^16,17^. OCAs frequently can topically clear tissue, and this procedure may be suitable for some surface imaging methods, such as OCT^18^. We require more efficient methods at deeper depths for deep imaging modalities like DOT and PAT to supplement the performance of OCAs. Therefore, for more effective clearing, complementary or alternate clearing approaches are needed. Recently, rapid, efficient, non-destructive, and effective agent-free optical clearing procedures have been developed for deeper tissue penetration^18–20^.

Ultrasound optical clearing can increase the light penetration depth by three mechanisms of hitting the surface of the tissue, creating gas bubbles inside the tissue, and producing a waveguide from the surface to the depth of the tissue. The first mechanism reduces light scattering by opening tissue pores. The second mechanism increases forward scattering by causing Mie scattering from the bubbles and the third process forms a channel inside the tissue that has a higher refractive index than the surrounding tissue and turns it into a light waveguide. In the Ref.^18^, for instance, ultrasonic radiation at a frequency of 1 MHz for 5 min increased light penetration depth into the human skin by up to 1.5 times, while the combination of ultrasonic, microdermabrasion, and agent-based techniques increased light penetration depth into the human skin by more than 2 times. Additionally, it was demonstrated that ultrasonic waves quicken OCA's spread^18^. In Ref.^19^, the light confinement in the tissue is caused by the gas bubbles created by the ultrasonic wave. In this method, bubbles are generated by high-intensity pulsed ultrasound at a desired depth and subsequently maintained by low-intensity continuous ultrasound during imaging. The laser can be tightly focused on a deeper imaging plane because of the bubble cloud's reduction of optical scattering and undesired changes in the incident photons' propagation direction. This phenomenon can be explained by the possibility that produced air bubbles that are as large as or larger than the light wavelength can act as Mie scatterers. The size of the bubble is dependent on the ultrasonic pulse frequency. For this aim, bubbles measuring around 25 μm in diameter were produced in Ref.^19^ using a 3MHz ring-shaped transducer. As a result, light is largely scattered in the forward direction. In the Ref.^20^, the interference of ultrasound waves (production of standing waves) in the tissue created a waveguide for the passage of light, which caused less scattering and confining of the light. The cross-section of the created waveguide was parallel to the surface of the tissue and its length extended deep (z-direction) into the tissue to a depth of about 8 mm. The width of the waveguide remained almost constant and about 1 mm from the surface to the depth. Also, the frequency of ultrasound waves was about 1.2 MHz.

Here, we have reduced light scattering and contained light using an ultrasonic waveguide. Agent-based and ultrasound-based clearing methods increase the penetration depth of light by reducing light scattering, while in our previous study^21^, a new method named Temporal Tissue Optical Clearing (TTOC) was presented, which increased light penetration depth in biological tissues by reducing both scattering and absorption. We theoretically and experimentally showed that at wavelength of 800 nm, 100 fs pulses penetrate 1.5 times more than 10 ns pulses in the gelatin-based phantom. The change of the probability of absorption and scattering in the ultra-short regime has been known in recent years. References^22,23^ have theoretically examined the interaction of ultra-short pulse with atomic and molecular targets, and their results show that absorption and scattering can be minimized at sufficiently short pulses (femtosecond and shorter pulses). In experimental studies, the results showed that the penetration depth of light is greater for picoseconds and shorter pulses compared to nanosecond and longer pulses. For example, in Ref.^24^, using ultra-short pulses the penetration depth of light increased (up to a few centimeters) and an interference pattern penetrated in the tissue without disruption for optical stimulation. While the interference pattern of long pulses is destroyed at short distances in tissue. In Ref.^25^, the use of ultra-short pulse lasers operating in the second near-infrared region (II-NIR) allows for increasing the penetration depth and providing spatial–temporal localization of radiation in mouse brain tissue. Also, the use of ultra-short pulses in underwater applications has also reduced the absorption and increased the length of underwater telecommunication links^26^. According to the reported results, ultra-short pulses with a high repetition rate can be a suitable alternative to short and long pulses to achieve greater penetration depth. In this paper, agent-based, ultrasound waveguide and temporal clearing methods are used to increase the light penetration depth in chicken breast tissue. Ultrasound method, by producing a waveguide, resolves the speed and depth limitations of conventional methods. Temporal method also by changing the interaction mechanism between the pulse and tissue, improves the optical clearing depth in addition to removing the speed limitation. It should be emphasized that at depth of tissue, the ultrasonic approach is more successful than the temporal method due to the broadening of the pulse width due to multi scattering during propagation. The combined optical clearing method introduced in this paper is low cost, fast, and efficient. Each form of optical clearing method, through various mechanisms, reduces the attenuation of light in the tissue. So, they can be complementary to increase the light penetration depth. Here, the greatest light penetration depth in chicken breast tissue (6.7 cm) has been reported by effectively combining three techniques. The use of this method increases the quality of optical imaging and diagnostic methods and can open a new window for researchers in this field.

## Results

**Figure 1:**
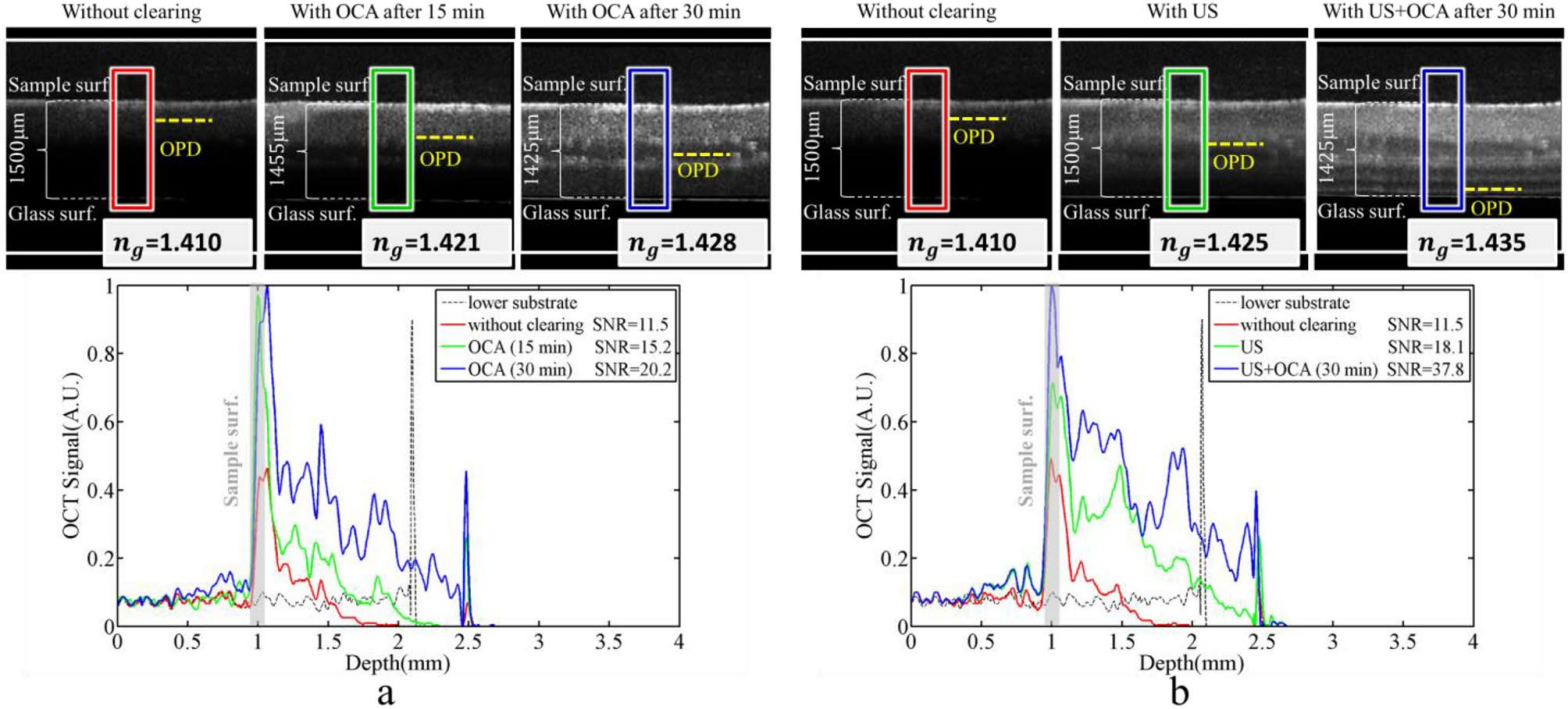
(**a**) OCT image and A-line signal of ROIs before (red line), for agent-based clearing after 15 min (green line) and after 30 min (blue line), (**b**) OCT image and A-line signal of ROIs for without clearing (red line), by ultrasound-based clearing (green line) and for combined clearing method by agent-based clearing after 30 min and ultrasound-based (blue line). OPD is the depth at which the OCT signal intensity reaches 1/e of its initial value. The OCT images were repeated for three points very close to each other on the sample, and a one-way ANOVA with Bonferroni post-hoc test was performed (*p* < 0.03) which shows that different groups of results are significantly different from each other.

The purpose of the tests performed in this paper is to introduce a set of techniques to achieve the greatest possible penetration depth of light in biological tissues. For this purpose, three methods of optical clearing, agent-based (immersion of chicken breast tissue in 75% glycerol solution), ultrasound waveguide (creating a waveguide using the interference of ultrasound waves) and temporal (reducing absorption and scattering using ultra-short pulses) have been used. To check the effect of clearing methods, OCT, fluorescence, and Beer-Lambert tests have been performed after applying the clearing procedure. In the OCT test, the image of the cleared tissue (by agent-based and ultrasound-based methods) is compared, and the greater imaging depth indicates the greater efficiency of the clearing method. In the fluorescence test, the amount of fluorescence signal has been measured using polymethine cyanine dye (LS-277) solution in ethanol. A more fluorescence signal, after tissue clearing, means more depth of light penetration in the tissue. Finally, in the Beer-Lambert test, the attenuation coefficient and light penetration depth are determined by measuring the light intensity before and after the light passes through the sample tissue. This is done in accordance with the Beer-Lambert relation. First, the results of optical clearing with 75% glycerol solution were checked before immersion and at 15 and 30 min after immersion of sample in the glycerol solution using the OCT system. Figure 1 shows the OCT images and regions of interest (ROIs) signal. Red color is selected for without optical clearing, green color is selected for optical clearing after 15 min, and blue color is selected for optical clearing after 30 min. The ROIs signals are the average of the A-scans values in OCT image. Also, signal-to- noise ratio (SNR) of OCT images was calculated in ROIs and written inside the figure. Over time, diffusion of glycerol inside the tissue gradually causes tissue water to get out of the sample. This leads to tissue shrinkage, removing the refractive index mismatches into the tissue and so increasing in light penetration depth. It should be noted that tissue shrinkage of the used samples led to decrease in their thickness less than 3% after 15 min and about 5% after 30 min of applying solution. The amount of tissue shrinkage is determined by measuring the distance between the tissue surface and the glass surface under the tissue in the OCT image. The light penetration depth in the OCT image is defined as the depth at which the signal intensity is 1/e of the top surface signal intensity. To calculate the optical penetration depth (OPD), the average A-scan signal vs. depth graph was fitted with a line and the penetration depth (yellow dashed line in the OCT images of Fig. 1) was determined at the point where the graph reached 1/e of its original value. Before clearing, the OCT image shows up to about 250 μm below the tissue surface, while 15 and 30 min after immersion in glycerol, the result shows up to a depth of 440 μm and 700 μm, respectively.

Next, we examine the tissue clearing using ultrasonic technique. In the presence and absence of a clearing agent, OCT imaging utilizing ultrasound-based optical clearing is displayed in Fig. 1b. According to the Fig. 1b, ultrasound-based clearing alone (green line) increases the depth of OCT imaging up to 600 μm. Also, the combination of ultrasound and agent-based methods (after 30 min) increases the depth of OCT up to 1.3 mm (blue line). After 15 min, the comparison of SNRs in Fig. 1 demonstrates that ultrasound-based clearing is more effective than agent-based clearing. Additionally, a particularly good improvement in the OCT image quality has been achieved by combining the two approaches. As a result, agent-based and ultrasound-based techniques can be complementary to one another, and simultaneously using them can improve optical imaging techniques. The TTOC approach was not examined using OCT test since access to an OCT system with an ultra-short source was restricted. Another useful piece of data that can be obtained from OCT images is the group refractive index (*n*_*g*_) for tissue. To measure *n*_*g*_, we first capture the OCT image exclusively from the bottom substrate (the glass surface), with no tissue present. The OCT image shows a shift in the peak that corresponds to the glass surface depending on whether the sample is present or absent. For determining the refractive index, the amount of shift (optical shift (OS)), and distance between surface and bottom peaks (optical thickness (OT)) are crucial. Finally using introduced method in Ref.^27^, *n*_*g*_ is obtained from *n*_*g*_ = OT/(OT − OS). The group refractive index of the sample in each mode of clearing is depicted in Fig. 1.

**Figure 2:**
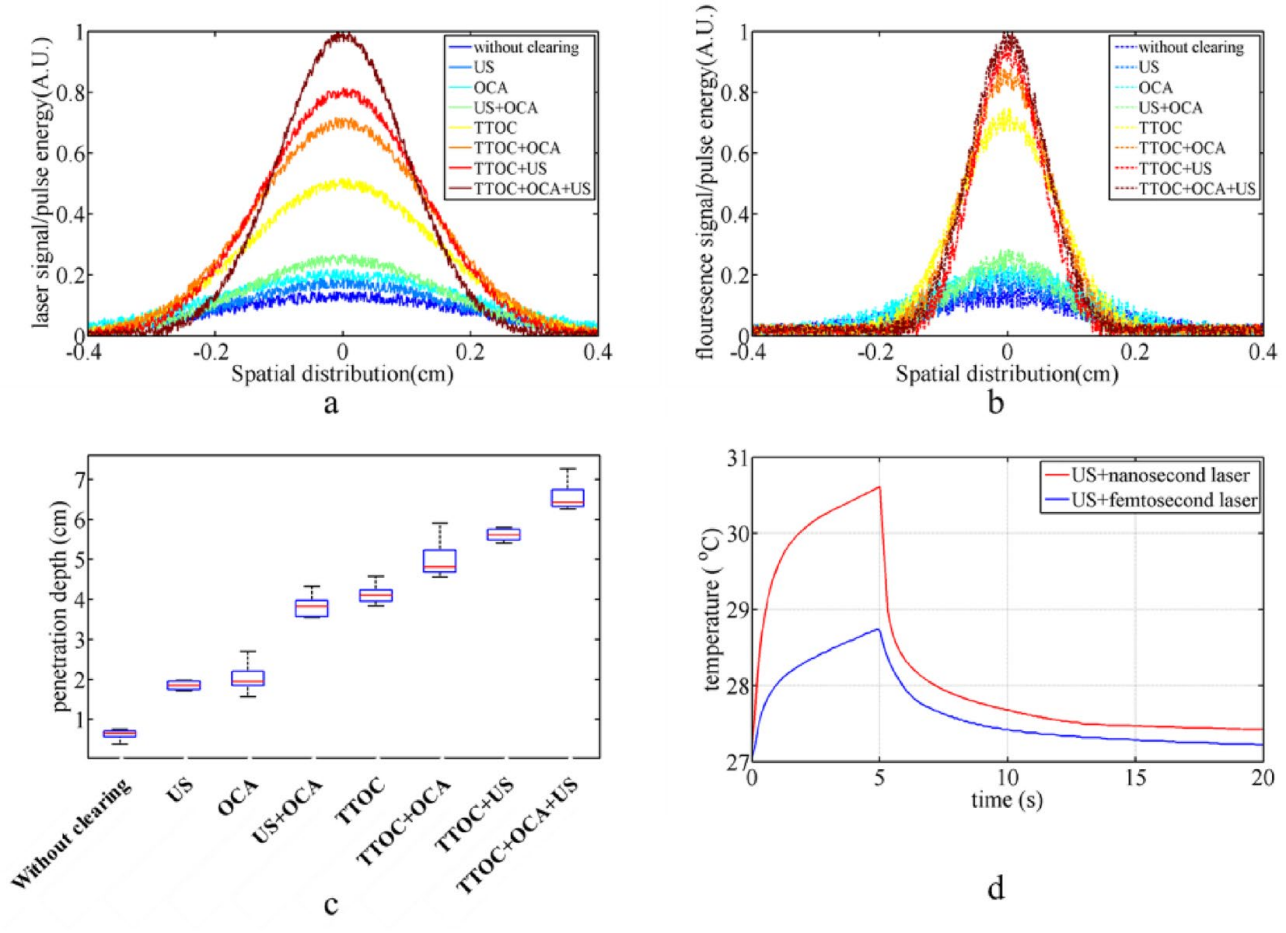
Spatial distribution of (**a**) received Laser light to CCD and (**b**) fluorescence signal per unit pulse energy for different clearing modes. (**c**) Light penetration depth for different modes of clearing obtained from Beer-Lambert test. The number of performing each of modes in part c is 5 times, and the error bars indicate the standard error. Statistical significance (*p* < 0.01) was determined by one-way ANOVA with Bonferroni post-hoc test. (**d**) It shows temporal variation of tissue temperature for the simultaneous use of US and TTOC methods.

In addition to the OCT image, the fluorescence test has been carried out to compare the impact of various clearing techniques on the depth of light penetration. In this test, light enters the sample, travels through it, and then absorbs in the infrared dye solution (LS-277 in ethanol), producing fluorescence radiation. A deeper penetration is indicated by a brighter fluorescence signal. Fluorescence test was used to validate the efficacy of all three optical clearing techniques (30-min agent-based, ultrasonic, and temporal) as well as their combined modes. This test monitors light transmission in addition to evaluating the fluorescence signal. Increased transmission indicates more effectively cleared tissue. Due to the dependence of the fluorescence signal on the number of incident photons, it is obvious that nanosecond laser with more energy and number of photons, can produce more fluorescence than femtosecond laser. But this comparison can be confusing. For proper comparison between different pulse widths in temporal optical clearing, the effect of the number of photons must be removed. Therefore, the fluorescence and transmitted laser signal in Fig. 2 are reported per unit pulse energy (fluorescence and transmitted laser signal normalized to the initial energy of the pulses). To calculate the laser energy, we first measure the laser power using a power meter and then calculate the energy by multiplying the power by the radiation time. Therefore, in the reported results, the difference in fluorescence test is only due to the different penetration depth in clearing methods, and the energy difference of the two nanosecond and femtosecond lasers has no effect on the reported signals. Figure 2a and b shows the spatial distribution of transmission of laser light and fluorescence signal per unit pulse energy on the CCD screen for three tissue clearing methods and their combined modes. The transmission light at wavelength of 800 nm and fluorescence light at wavelength of 821 nm are separated using a long pass filter at 810 nm ± 8 nm. Only the fluorescence light was able to reach the CCD when the filter was in place; when it wasn't, both types of light were able to reach the CCD. It is evident that when all three techniques were used at once, the greatest transmitted light and fluorescence signal were produced. The fluorescence signal in Fig. 2b is around ten times stronger than the fluorescence signal in the absence of clearing. Additionally, the findings of the fluorescence test demonstrate that, in chicken breast tissue, the temporal clearing method by itself is more effective than the other two clearing techniques. Consequently, when compared to alternative methodologies, the signals associated with the TTOC method have increased dramatically in Fig. 2a and b. Another significant finding in Fig. 2b is that the enhancement factor of the fluorescence signal reduced because of improving the depth of light penetration and reducing absorption and scattering. For instance, the fluorescence signal increased around seven times when the TTOC approach was employed alone, but the fluorescence signal increased ten times when three methods were combined. This outcome demonstrates that the fluorescence signal cannot be linearly increased by combining the three approaches. Moreover, the peak signal-to-noise ratio (PSNR) has been calculated for Fig. 2a and b. PSNR is an expression for the ratio between the maximum value of a signal and the average of distorting noise in terms of the logarithmic decibel scale. In the transmission signal, the PSNR values increase from 16.7 dB (without optical clearing) to 29.2 dB (all three optical clearing modes); in the fluorescence signal, the PSNR changes from 12.8 dB (without optical clearing) to 24.7 dB (all three optical clearing modes). Finally, the results of the Beer-Lambert test for the comparison of three clearing methods are presented. In this test, the light intensity is measured before and after passing through the tissue, and the effective attenuation coefficient is calculated according to Beer-Lambert equation. It should be noted that nanosecond diode laser and femtosecond Ti: Sapphire laser at wavelength of 800 nm were used for both fluorescence and Beer-Lambert tests. Considering that the light penetration depth for ballistic photons is defined as 1/μ_t_ (where μ_t_ is the total attenuation coefficient and the sum of absorption and scattering coefficients of tissue), it is possible to compare the penetration depth in different clearing modes. Figure 2c shows the light penetration depth measured in chicken breast tissue. As expected, the combination of three methods produced the highest penetration depth, which is 10 times the penetration depth for the tissue without clearing.

To analyze the change that occurs in the optical coefficients of the tissue, it should be noted that the light penetration depth for ballistic photons is equal to the inverse of the total attenuation coefficient. In the ultrasound optical clearing method, the change is only caused by the reduction of the scattering coefficient and the absorption coefficient remains constant^20^. Based on the penetration depth results in Fig. 2c, the scattering coefficient in the ultrasound method is 0.3 times compared to the initial state. In agent-based method, as the solution spreads in the tissue, absorption, and scattering change, but these two are indistinguishable from each other. Therefore, it can be said that the μ_t_ becomes 0.27-fold in agent-based optical clearing. Compared to the OCT test, it can be said that transmission-based tests such as Beer-Lambert and fluorescence are more sensitive to tissue shrinkage phenomenon. Therefore, the changes in the attenuation coefficient and the penetration depth after clearing solution treatment are greater in the transmission-based tests. In the combination of ultrasound and agent-based methods, the μ_t_ becomes 0.15 times. Also, The TTOC method reduces both absorption and scattering, and the μ_t_ becomes 0.14 times. The greatest reduction of the attenuation coefficient occurs in the simultaneous use of three methods, which becomes 0.1-fold.

An intriguing aspect in Fig. 2 illustrates how combining different clearing techniques affects clearing efficiency. When we utilize the Ultrasound (US) approach alone, as shown in Fig. 2c, the penetration depth is increased by 1.2 cm. Additionally, the light penetration depth increases by 1.4 cm when using OCA alone; however, the penetration depth increases by approximately 3.2 cm (greater than the total of the effects of OCA and US methods) when using OCA and US methods together. This means that OCA and US methods can reinforce each other’s effects while this issue is different in the case of TTOC in combination with other methods. When the TTOC is used alone, 3.5 cm is added to the light penetration depth, but when it used in combination with the OCA and US, the penetration depth increases by 4.4 cm and 5 cm respectively (less than the sum of the effects of methods). This result shows that although combining TTOC with US and OCA improves the penetration depth, it slightly reduces the efficiency of the TTOC. However, the highest penetration depth (in Fig. 2c) and the highest fluorescence signal (in Fig. 2b) were obtained in the combination of three methods (6.7 cm). Also, the comparison between different groups of results (OCA, US and TTOC) in Fig. 2c using the Bonferroni test shows that for all comparisons *p* < 0.01 and the results of different groups are significantly different from each other.

One problem with the results is that, in Fig. 2, the combination of TTOC and US produces a better result than the combination of TTOC and OCA, even if the clearing result with OCA alone (after 30 min) is better than US. There could be two important components to this event. First, because glycerol diffuses into the tissue, a sizable portion of the interstitial fluid is made up of it. This changes the optical characteristics of the tissue. Presumably, the TTOC approach will behave differently in the presence of glycerol and will change the tissue's optical characteristics compared to when glycerol is absent. We studied this phenomenon separately and investigated the light attenuation in glycerol solution with different percentages using the same two lasers (nanosecond and femtosecond) in the Beer-Lambert test arrangement. For 20% glycerol, when we used a femtosecond laser instead of a nanosecond laser, the attenuation of the solution reduced by 48%, while in a 75% glycerol solution, the percentage of attenuation reduction was 27%. This study shows that the presence of more glycerol reduces the efficiency of the TTOC method. The second factor is the dehydration and variation of the refractive index of tissue after solution treatment, which changes the optical properties of the tissue to reduce the efficiency of TTOC^28^. Controlling the tissue temperature is crucial when using TTOC and US techniques at the same time. We subject a tiny sample of chicken breast tissue (5 mm × 5 mm × 2 mm) to ultrasonic waves and laser pulses to measure the temperature of the tissue. An ultra-fast thermocouple with a response time of 20 ms and an accuracy of 1 °C was used to measure the temperature. After 5 s of ultrasonic and laser pulse irradiation, Fig. 2d displays the tissue's temperature in terms of time. It is evident that the combination of ultrasonic waves and nanosecond pulses results in the maximum temperature rise. However, since the temperature rise is less than 3.5 °C, it can be argued that tissue damage is not caused by these optical clearing techniques.

## Conclusion

In this work, the light penetration depth in chicken breast tissue was measured using three different tissue clearing techniques: agent-based, ultrasound-based, and a novel technique known as temporal clearing. While the temporal method reduces both light scattering and absorption in the tissue, agent-based and ultrasound-based methods reduce light scattering in the tissue^21^. To compare the optical clearing techniques and determine the depth of light penetration in the tissue, OCT imaging, fluorescence, and Beer-Lambert tests were employed. The temporal approach outperforms the other two methods in terms of efficiency, according to the results. The results of Ref.^24^, where the interference pattern penetrated several centimeters deep into the tissue, are consistent with increasing the light penetration depth in the tissue by using the temporal technique. Also, the results of ultrasound-based clearing are consistent with the results of Ref.^20^, where the depth of light penetration in the agar phantom was increased by using an ultrasound waveguide. From the results, it can be concluded that the combination of methods reduces the efficiency of the temporal method. While ultrasound-based and agent-based methods do not have a negative effect on each other's performance. The maximum depth of light penetration in chicken breast tissue was obtained about 6.7 cm by simultaneous use of three clearing methods, which is 10 times more than the penetration depth in tissue without clearing. Compared to the other papers, such as Ref.^18^ (2-folds increase of light penetration depth from about 600 µm to about 1200 µm in human skin), Ref.^19^ (up to 6-folds increase of light penetration depth from 69 to 437.4 µm in biological phantoms), and Ref.^24^ (2-folds increase of light penetration depth from less than 1 mm to about 2 mm in chicken breast tissue), here, a new record for increasing light penetration depth in tissue is reported. The combination of methods reviewed in this paper can improve the quality of imaging in optical imaging methods such as OCT, photoacoustics, fluorescence, and time-gate DOT. Given that the strategies described here can significantly improve the quality of medical imaging when applied to various optical imaging techniques. For instance, combining the three optical clearing approaches will advance this technology and enhance cellular and subcellular OCT imaging. By raising the number of confined photons in the depth of tissue, it will also boost the depth and resolution of deep imaging techniques like DOT and PAT. There are difficulties with the collection of approaches described here to improve the depth of light penetration in the tissue. For instance, the application of TTOC, which decreases light absorption and scattering, can lower imaging contrast since optical imaging methods rely on the absorption or scattering of light in the tissue. Reduction of scattering can decrease the contrast of scattering-based methods such as OCT and reduction of absorption can decrease the contrast of absorption-based methods such as PAT. Therefore, these methods should be applied with caution in the interpretation of the received images because they are targeted for the analysis of in-depth structures for which contrast is improved. In addition, the usage of this set of techniques for in vivo imaging has the challenge of arranging ultrasound modules. In the imaging of some tissues, it is difficult to place ultrasound modules in a surrounding manner. However integrated OCT-PAT systems are described in literature, even in endoscopic mode; see for example^29^.

## Materials and methods

In this paper, three different clearing methods are used to increase the penetration depth of light in chicken breast tissue. The details of the experimental setup, materials and tools used to perform multimodal clearing are described in this section.

### Experimental setup to determine the depth of light penetration

**Figure 3:**
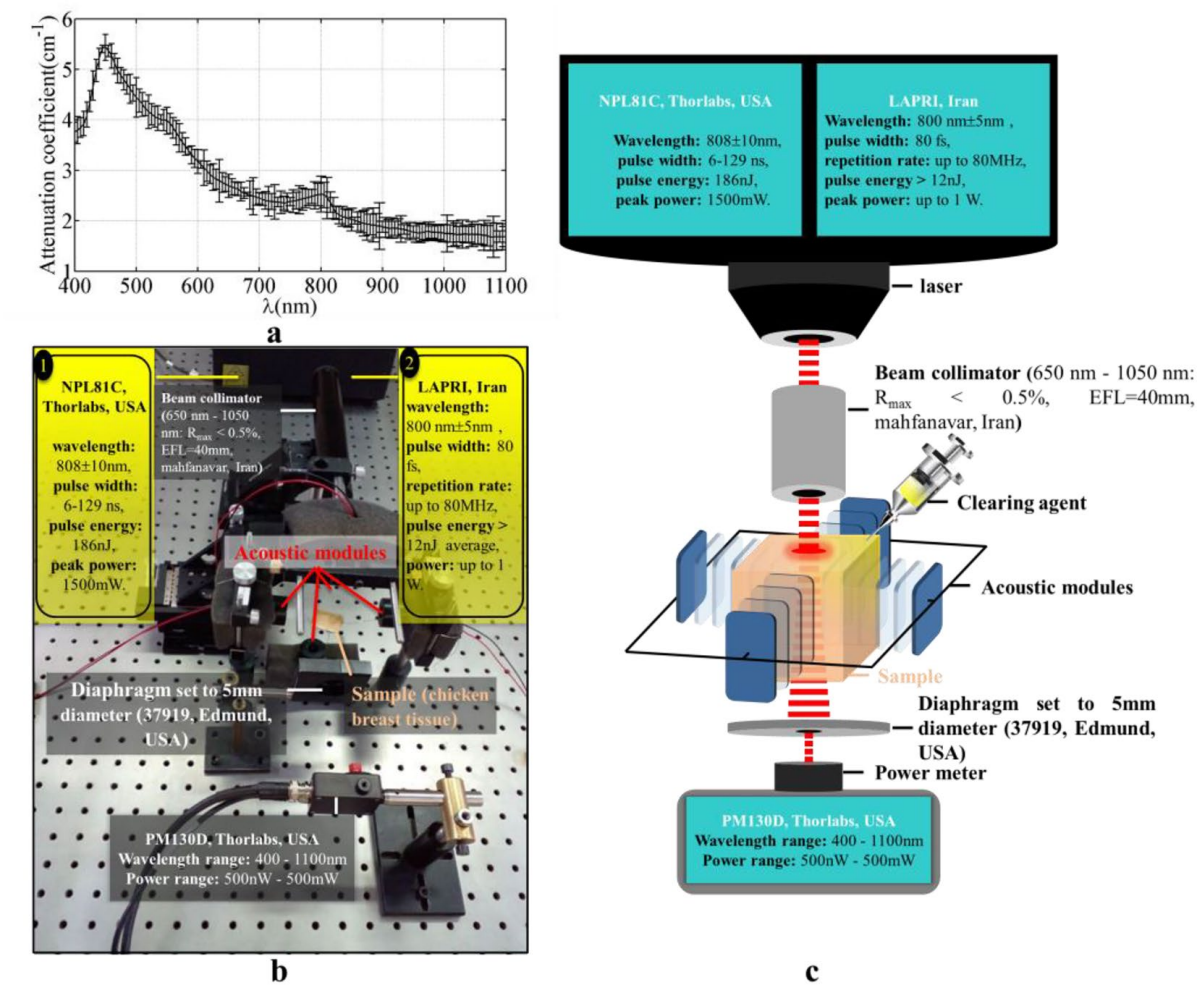
(**a**) Attenuation characteristics of chicken breast tissue (averaged for 4 tissue samples), the error bars indicate the standard error. (**b**) Experimental and (**c**) schematic setup of Beer-Lambert test.

45-day Fresh chicken breasts were purchased in a specialized chicken sales center (Tehran, Iran). Several similar samples of chicken breast tissue were prepared uniform size, shape, and weight (4 cm × 3 cm × 0.15 cm, 2.25 ± 0.05 g) to investigate clearing methods. The size of the samples was measured by a caliper with accuracy of 0.02 mm. Optical attenuation of prepared samples was determined by a custom modified Double Beam UV–Vis Spectrophotometer (UV-2100, BRAIC, China). Tungsten source is used in this system and its working wavelength range is from 400 nm to about 1100 nm. Figure 3a shows the attenuation of light in the range of visible and near infrared in chicken breast tissue. The ridges in the attenuation graph around 450 nm and 550 nm can be due to the presence of hemoglobin in the tissue, and the ridge around 800 nm is an isosbestic point for oxy- and deoxyhemoglobin absorption^30^. It should be noted that the actual peaks of the mentioned compounds are about 20–25 nm lower than what is seen in the figure, but the calibration of the UV–VIS spectrometer has caused this shift. Three tests such as Beer-Lambert, OCT imaging and fluorescence have been used to check the efficiency of clearing methods.

The experimental and schematic setup of the Beer-Lambert test can be seen in Fig. 3b and c respectively. In this setup, two different laser pulse widths are used to check the temporal clearing. The first is the nanosecond diode laser (NPL81C, Thorlabs, USA) and the second is the femtosecond Ti: Sapphire laser (LAPRI, Iran). Also, a power meter (PM130D, Thorlabs, USA), was used to determine the power of the beam before and after passing through the sample. Beer-Lambert's equation expresses the relationship between the transmitted and incident light intensity for ballistic photons as follows^31^:

1 $$\frac{I}{{I_{0} }} = \exp ( - \mu_{t} l),$$

Where *I*_*0*_ is the intensity of the laser beam before entering the sample, *I*, is the intensity of transmitted laser beam, *μ*_*t*_, is the attenuation coefficient (The sum of absorption coefficient, *μ*_*a*_, and scattering coefficient, *μ*_*s*_), and *l*, is the path length (sample thickness). We measured *I*/*I*_0_ by power meter and calculated *μ*_*t*_ from Eq. (1). Light penetration depth is defined as the inverse of the effective attenuation coefficient (1/μ_eff_). While for ballistic photons, the penetration depth may be calculated as 1/(µ_s_ + µ_a_) and for diffused photons as 1/(3µ_a_(µ_a_ + µ′_s_))^1/2^, respectively, which, µ′_s_ the reduced scattering coefficient ($\mu \prime_{{\text{s}}} = \, \mu_{{\text{s}}} \left( {{1 }{-}g} \right)$)^31^. Because direct photons are detected in the Beer-Lambert test, the penetration depth in this test is measured for ballistic photons. Beer-Lambert test can be used to determine the depth of penetration in all the clearing methods used in this paper. It is noteworthy that according to the characteristics of the used lasers, the average power of ns pulses is about 7.5 × 10^–4^ W and the average power of fs pulses is about 8 × 10^–6^ W. Therefore, nanosecond pulses create more heat in the tissue, which confirms the results shown in Fig. 2d. Another method used to check the depth of light penetration is OCT imaging. For this purpose, Swept-source OCT system (DRI, Topcon, USA) was used which has a continuous source with a central wavelength of 1050 nm. OCT imaging has been used to determine the penetration depth for agent-based and ultrasound-based clearings. To compare the quality of OCT images, the SNR has been used which is calculated as the ratio of the average image intensity to its standard deviation for ROIs^32^.

**Figure 4:**
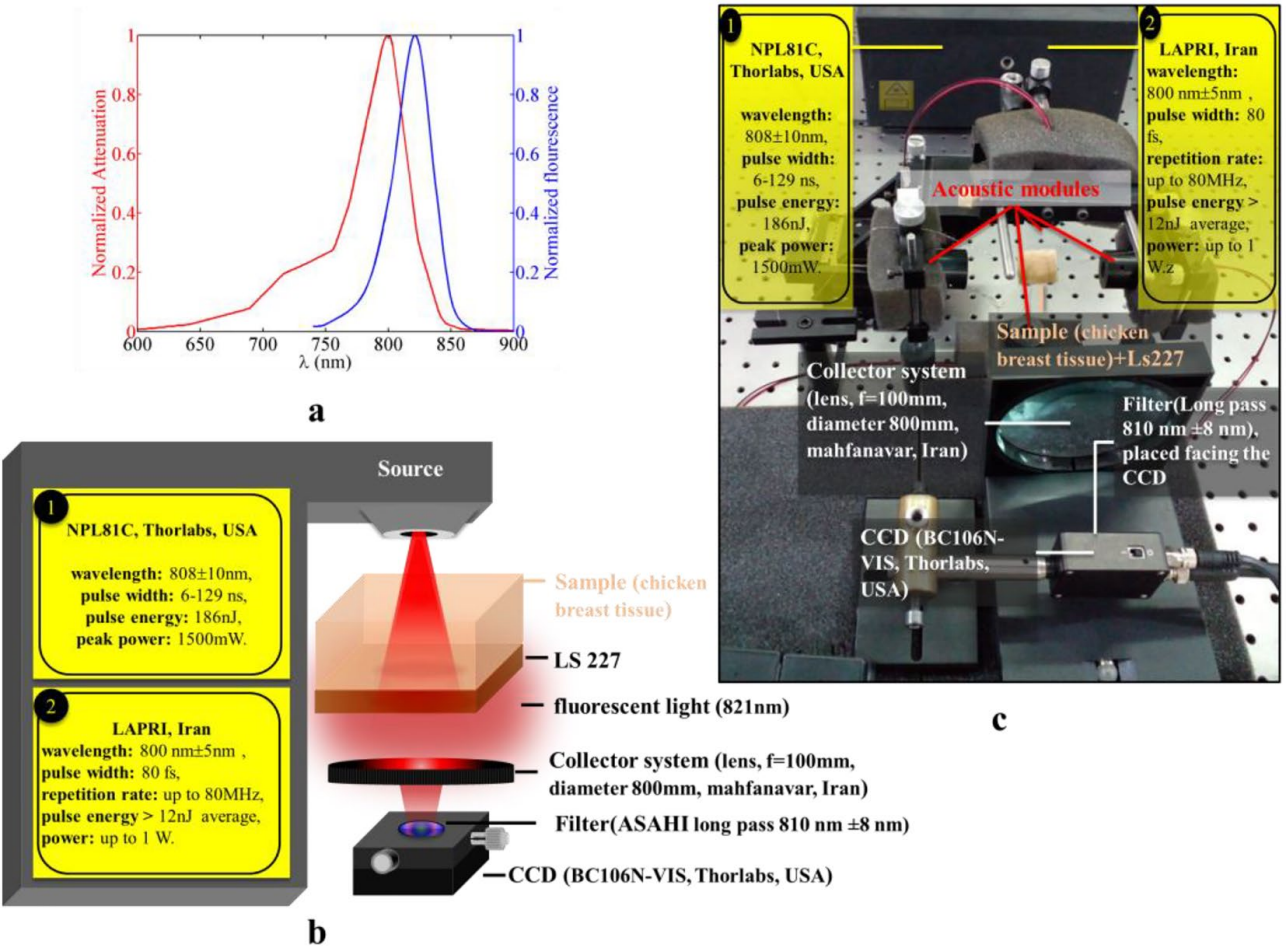
(**a**) Normalized absorption and fluorescence signal of LS-277/ethanol solution, (**b**) Schematic and (**c**) experimental setup of fluorescence test.

The third method of checking the depth of light penetration is the fluorescence test, which is detailed in Fig. 4. For this purpose, we used a solution of near infrared dyes. One of the common fluorophores at wavelength of about 800 nm is polymethine cyanine dye LS-277, which can be purchased at a low price. Although LS-277 (Xchem, Iran) was purchased for the tests of this paper, its synthesis conditions are according to method presented in Ref.^33^.

We smeared the back surface of the tissue with a solution containing 10 g of LS-277 and 10 ml of 70% ethanol solvent. Figure 4a shows the absorption and fluorescence spectrum of the prepared solution, which was measured using a Double Beam UV–VIS Spectrophotometer. The maximum absorption of the solution is at the wavelength of 800 nm, while the fluorescence light of the solution is at the wavelength of 821 nm. The lasers used in this test are nanosecond diode laser and Ti: Sapphire femtosecond laser. Separation of transmitted laser light from fluorescence light was done using ASAHI long pass frameless filter that has 99.5% transmittance at wavelengths above 810 nm. This circular shape filter has a diameter of 25 mm. The fluorescence signal intensity and the transmitted laser light are measured using a CCD beam profiler (BC106N-VIS, Thorlabs, USA). If the depth of light penetration in the sample increases due to clearing, the intensity of the fluorescence signal increases. Figures 4b and 4c show the schematic and experimental setup of the fluorescence test.

### Agent-based clearing

**Figure 5:**
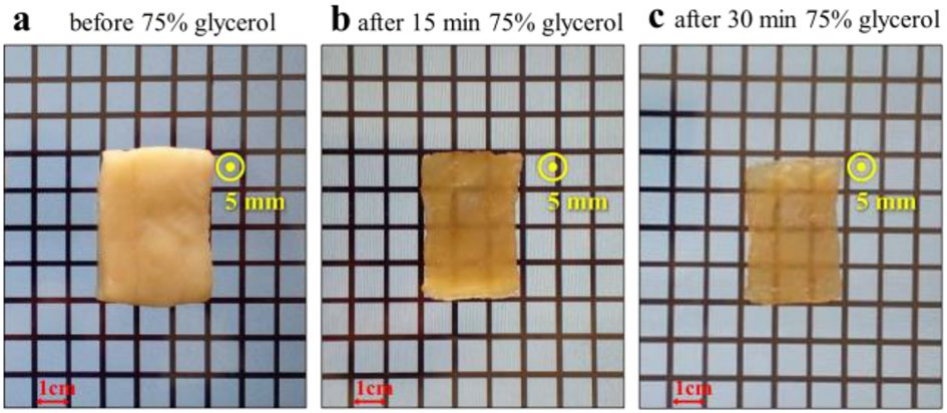
Chicken breast tissue (**a**) before clearing, and (**b**) after 15 min and (**c**) after 30 min immersion in 75% glycerol solution.

Factors such as toxicity, dehydration process, diffusion properties, and skin inflammation are influential in determining the clearing of OCA. Glycerol is one of the common OCAs, which is used in this paper by volume percentage of 75%. The samples were immersed in the solution for 15 min and 30 min^6^. The important point in agent-based clearing is sample shrinking and reducing its dimensions. Therefore, after the immersion process, the dimensions of the sample were measured, and the new dimensions were used in the penetration depth calculations. The immersion process after 15 and 30 min approximately decreases the thickness of tissue 60 and 100 μm, respectively. Figure 5 shows a sample of chicken breast tissue before and after agent-based clearing by glycerol.

### Ultrasound-based clearing

**Figure 6:**
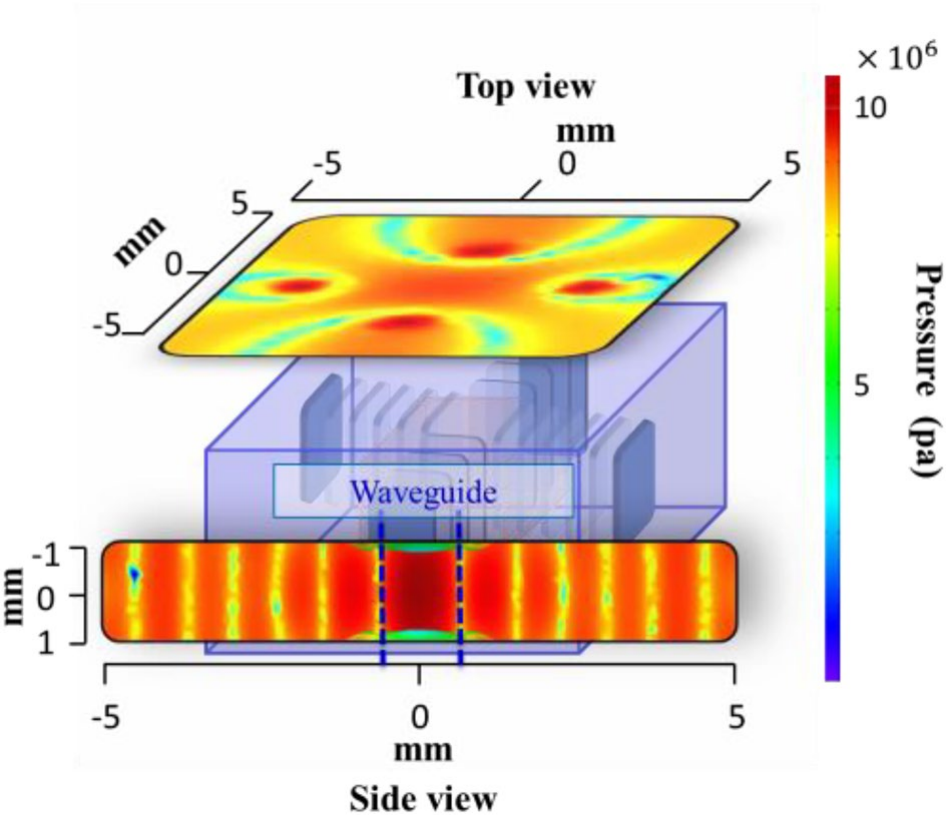
Modeling the interference of ultrasound waves in tissue in COMSOL software.

The main idea of ultrasound-based optical clearing in this paper is to create a waveguide for the passage of light from the surface to the depth of the tissue using ultrasound standing waves. To form this waveguide, the modules must radiate the ultrasound wave perpendicular to the tissue from around. Therefore, the best arrangement using 4 ultrasound modules can be two by two facing each other around the sample. Ultrasound modules with a frequency of 1.7 MHz (24V DC-1200 mA-AE1322) are cylindrical in shape and have a circular Effective Radiating Area (ERA) equal to 7 cm^2^. The wave produced by the modules is continuous and the weight of each module is about 300 g^20^. To determine the shape of the standing waves on the sample, the interference of ultrasound waves, and the simulation of ultrasound-based optical clearing along with agent-based optical clearing, the Finite Element Method (FEM) was used in commercial software (COMSOL, Inc.), using a coupled multi physics simulation including solid mechanics and pressure acoustics. In this software, for the ultrasound module structure the Navier–Stokes equations and for the pressure acoustics the Helmholtz equation are solved^20^. It should be noted that tissue shrinkage due to OCA treatment is considered in COMSOL simulations. The placement of ultrasound modules and modeled interference pattern of ultrasound waves are shown in Fig. 6. Material and geometrical properties for simulation are provided in Table1. The central area of this interference pattern has high refractive index, which can be a waveguide for light propagation. Normally, the refractive index of chicken breast tissue is 1.410. The center of the waveguide region has a maximum refractive index of 1.425. The width of the waveguide is about 1 mm. This method causes the light to be confined inside the waveguide and therefore reduces the scattering of light around. The important point in creating a waveguide is that high pressure areas are not constant and change their place with low pressure areas. The speed of changing high-pressure and low-pressure areas is of the order of the sound speed in the medium and much lower than the speed of light. The created waveguides only exist for a limited period (about a few μs) and then they give their place to low-pressure areas and this process is repeated. As a result of the periodic production of the waveguide, the scattering of light is reduced, and the average transmitted light power in the waveguide area recorded by the power meter increases.

**Table 1:** parameters used in COMSOL simulation.

Ultrasound module (cylindrical)	
Material	Piezoelectric PZT—5A
Density	7500 kgm^−3^
Radius	15 mm
Height	25 mm
Simulation domain	
Dimension	40 mm × 30 mm × 2 mm
Medium density (tissue)	1250 kgm^−3^
Speed of sound	1580 ms^−1^

### Temporal tissue optical clearing

**Figure 7:**
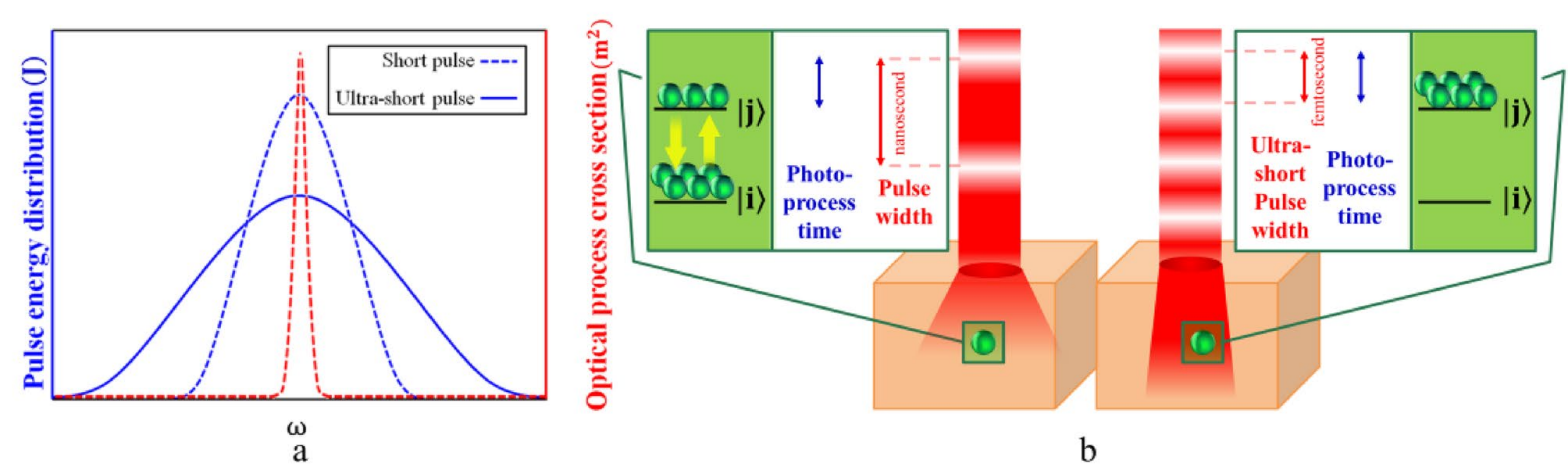
(**a**) Changing the pulse energy distribution by changing the pulse width. (**b**) The difference between ultra-short pulses and other pulses in the saturation of the target material.

There are two methods for reducing absorption and scattering in the range of ultra-short pulses. In the first mechanism, altering the laser pulse width will alter the distribution of the pulse energy across various frequencies, which will alter how the pulse interacts with matter. The frequency distribution of the pulse energy and the attenuation cross-section of the matter are superimposed to define the probability of light absorption and scattering in the medium, and changing the pulse width in the ultra-short regime can alter the attenuation characteristics^22^. A broadening of the pulse energy distributionA in the side frequencies results from decreasing the pulse width, as schematically seen in Fig. 7a. Therefore, a smaller portion of the pulse energy is concentrated in the peak of matter attenuation. Figure 7a demonstrates how well this process works to lessen attenuation in materials that exhibit an attenuation peak at the laser wavelength. In the second process, the target matter's attenuation factors are saturated by the high intensity of the ultra-short pulses, which lowers the attenuation. With increased intensity and laser repetition rate, this process reduces attenuation in target materials, and its efficacy rises^30^. The attenuation reduction's saturation mechanism is depicted in Fig. 7b. Optical process time (the time required for absorption or scattering by the attenuator) is inversely proportional to the spectral width of attenuation cross section^34^. So, in the case of chicken breast tissue, it can be about a few femtoseconds. In long and short pulses (longer than nanosecond), the pulse width is much greater than the optical process time. Therefore, the attenuation factors can be stimulated many times and return to the initial state before the pulse has passed completely. But in the case of ultra-short pulses (shorter than ps), the front part of the pulse causes saturation of the attenuator due to its high intensity, and the end part of the pulse passes completely before the attenuator returns to its initial state. The effectiveness of each mechanism in the temporal method is unknown and requires further investigation. Many studies have been done on the saturation mechanism in the field of Electromagnetically Induced Transparency (EIT). Works on EIT describe single-band atomic and molecular systems^35^. In the case of tissue, which is a condensed medium, due to the presence of many absorption bands, absorption and scattering cannot be eliminated at saturation, but their decrease can be expected.

The three methods reviewed in this section for tissue clearing can affect each other's performance. For example, by injecting OCA, the frequency distribution of the attenuation in the sample will change, and as a result, the performance of the temporal clearing will be affected. Also, creating a standing wave inside the tissue affects the distribution of OCAs and their performance. We have checked all the combined modes of clearing methods to reveal the effect of clearing methods on each other.

## Data Availability

Data underlying the results presented in this paper are not publicly available at this time but may be obtained from the authors upon reasonable request, e-mail: m_ansari@sbu.ac.ir.
